# Electrospun self-emulsifying core-shell nanofibers for effective delivery of paclitaxel

**DOI:** 10.3389/fbioe.2023.1112338

**Published:** 2023-01-19

**Authors:** Ruiliang Ge, Yuexin Ji, Yanfei Ding, Chang Huang, Hua He, Deng-Guang Yu

**Affiliations:** ^1^ Department of Outpatient, The Third Affiliated Hospital, Naval Medical University, Shanghai, China; ^2^ School of Materials and Chemistry, University of Shanghai for Science and Technology, Shanghai, China; ^3^ Sinopec Shanghai Engineering Co., Ltd., Shanghai, China

**Keywords:** self-emulsifying, coaxial electrospinning, poorly water-soluble drug, drug delivery, medicated nanofibers

## Abstract

The poor solubility of numerous drugs pose a long-existing challenge to the researchers in the fields of pharmaceutics, bioengineering and biotechnology. Many “top-down” and “bottom-up” nano fabrication methods have been exploited to provide solutions for this issue. In this study, a combination strategy of top-down process (electrospinning) and bottom-up (self-emulsifying) was demonstrated to be useful for enhancing the dissolution of a typical poorly water-soluble anticancer model drug (paclitaxel, PTX). With polyvinylpyrrolidone (PVP K90) as the filament-forming matrix and drug carrier, polyoxyethylene castor oil (PCO) as emulsifier, and triglyceride (TG) as oil phase, Both a single-fluid blending process and a coaxial process were utilized to prepare medicated nanofibers. Scanning electron microscope and transmission electron microscope (TEM) results clearly demonstrated the morphology and inner structures of the nanofibers. The lipid nanoparticles of emulsions after self-emulsification were also assessed through TEM. The encapsulation efficiency (EE) and *in vitro* dissolution tests demonstrated that the cores-shell nanofibers could provide a better self-emulsifying process int terms of a higher EE and a better drug sustained release profile. Meanwhile, an increase of sheath fluid rate could benefit an even better results, suggesting a clear process-property-performance relationship. The protocols reported here pave anew way for effective oral delivery of poorly water-soluble drug.

## 1 Introduction

Solubility of drug is one of the most important standpoints for developing drug delivery systems (DDSs) for oral administration, the most popular and convenient route for the patients (Khan et al., 2015; Lv et al., 2021; Zhang et al., 2022a; Ejeta et al., 2022). However, the number of drug candidates with low solubility and high permeability (BCS Class II medicines) is always increasing, and the high water-soluble and permeable drug candidates occupy only 8% in pharmaceutical industry (Ortega et al., 2020; Köse et al., 2021; Ning et al., 2021; Chen et al., 2022a; Yu and Zhao, 2022). Thereby, the poor solubility of drugs pose a long-existing and difficult challenge to the researchers in the fields of pharmaceutics, bioengineering and biotechnology (Butreddy et al., 2021; Cai et al., 2021; Feng and Hao, 2021; Salerno and Netti, 2021; Sultana et al., 2021; Obeidat and Al-Natour, 2022). Many “top-down” and “bottom-up” nano fabrication methods have been exploited to provide solutions for this issue (Esim and Hascicek, 2021; Tabakoglu et al., 2022; Tang et al., 2022a; Vega-Vásquez et al., 2020).

**FIGURE 1:**
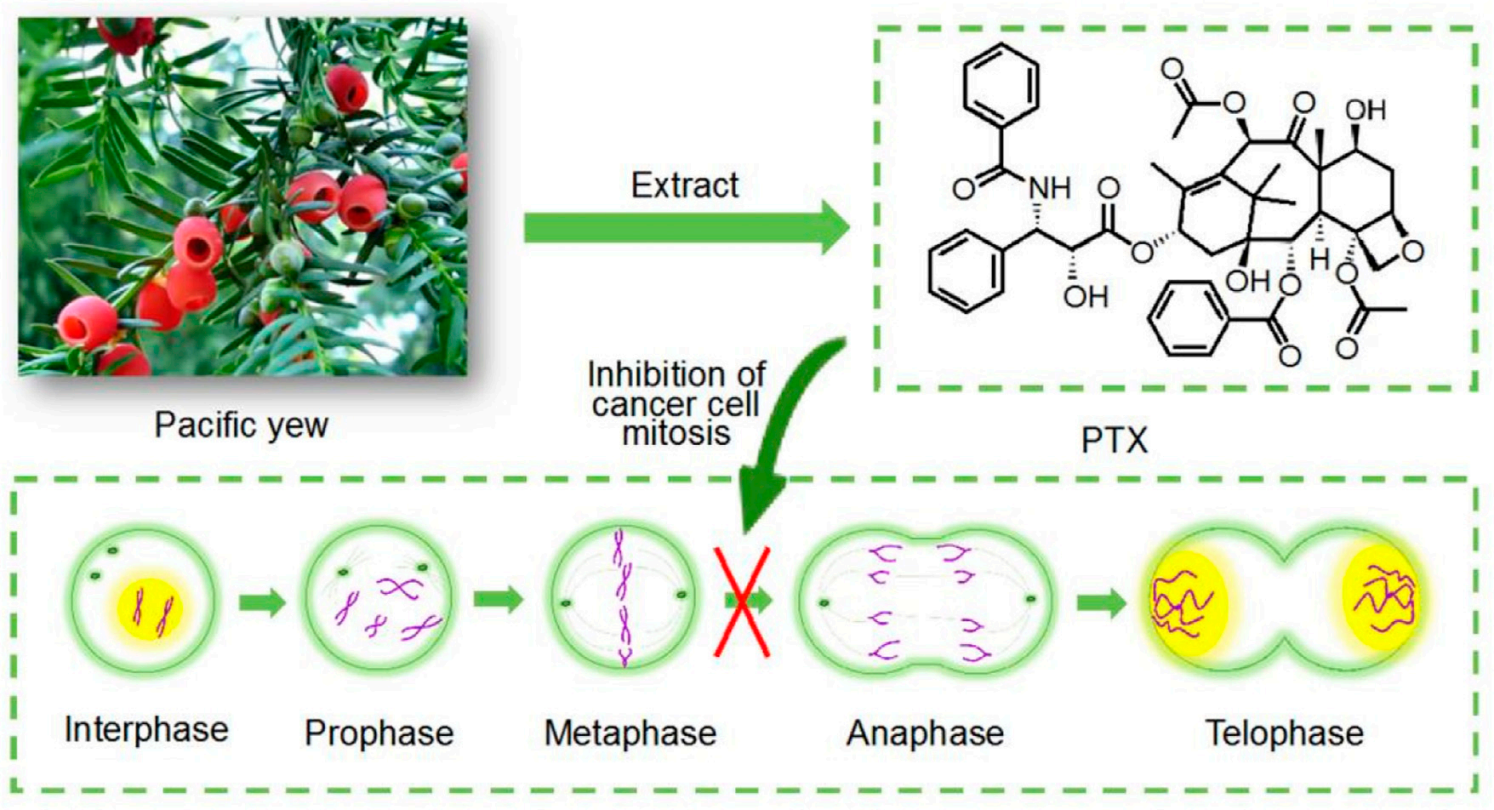
Mechanism of paclitaxel action.

Paclitaxel (PTX) is one of the most representative anticancer drugs, and it is the most excellent natural anticancer drug (Markman and Mekhail, 2002). In 1963, Wani and Wall first isolated the crude extract of paclitaxel. Due to the low content of active components, it was difficult to purify it. It was not until 1971 that the chemical structural formula of paclitaxel was determined (Martin, 1993; Zhang et al., 2013). Clinically, Taxol (PTX) has a good anti-tumor effect, especially for ovarian cancer, uterine cancer and breast cancer with high incidence rate of cancer (Zhang et al., 2013). It is considered to be one of the most effective anticancer drugs in the next 20 years. PTX has broad-spectrum anti-tumor activity. It is a drug that stabilizes microtubules and selectively destroys microtubule dynamics, thus inducing mitotic arrest leading to cell death (Figure 1). Because of its strong anti-tumor activity, PTX is often used in the treatment of esophageal cancer, bladder cancer, prostate cancer, cervical cancer, gastric cancer, head and neck cancer, endometrial cancer, oligodendrocytoma and testicular cancer (Martin, 1993). Although PTX and its analogues play an important role in conventional cancer chemotherapy, the development of intravenous PTX is difficult due to its poor solubility in water. Meanwhile, it has been reported that PTX injection can cause severe reactions such as bronchospasm and hypotension (Zhang et al., 2013). Therefore, PTX has been widely exploited as the model of poorly water-soluble drug in solving the issues of effective oral administration.

Self-emulsifying drug delivery system (SEDDS), as a kind of drug delivery system, has a history of more than 10 years (Agrawal et al., 2015; Khan et al., 2015). Self-emulsifying tablets have been used in clinical medicine since the 1990s (Truong et al., 2016). The advantage of this DDS is that it can not only form spontaneously absorbed small microemulsion through the kinetic energy generated by gastrointestinal peristalsis, but also can be administered through the simplest way of oral administration (Zaghloul et al., 2019; Li et al., 2021; Miar et al., 2021), and it can also encapsulate poorly water-soluble drugs to make it more stable (Patel and Sawant, 2009; Chouhan et al., 2015; McClements, 2015).

The development of DDS also stimulates the development of raw material selection and the introduction of advanced techniques as pharmaceutical methods (Feng and Hao, 2021). Electrospinning is one of the powerful methods for continuous production of drug loaded nanofibers (Xue et al., 2019; Liu et al., 2022a; Sivan et al., 2022b; Cao et al., 2022; Han et al., 2022; Lv et al., 2022), and electrospun nanofibers have been demonstrated their potential applications in almost all kinds of scientific fields such as energy, environment, medical and food (Pang et al., 2021; Jiang et al., 2022a; Li et al., 2022a; Bai et al., 2022; Xu et al., 2022; Yu et al., 2023). As a top-down technology, electrospinning is famous for its simplicity, efficiency and flexibility in direct preparation of nanofibers (Du et al., 2022; Huang et al., 2022; Yao et al., 2022). At present, there are many researches using electrospinning as a tool to construct medicated nanofibers (Huang et al., 2021; Peng et al., 2021; El-Shanshory et al., 2022; Hameed et al., 2022). These electrospun active nanofibers have been demonstrated to be useful for providing all kind of drug release profiles, such as pulsatile release, sustained release, delayed release, biphasic release, targeted release in a direct manner (Sivan et al., 2022a; Wang et al., 2022a; Jiang et al., 2022b; Liu et al., 2022b; Wang et al., 2022b; Zhao et al., 2022); and also for tissue engineering, wound dressing, and other regeneration medicines during the past two decades (Zhang et al., 2021a; Brimo et al., 2021; Zhao et al., 2021b; Chen et al., 2022b; Guo et al., 2022; Mosallanezhad et al., 2022; Shen et al., 2022; Sun et al., 2022).

However, there is very limited reports on the research to combine electrospinning with self-emulsifying methods to develop new types of drug delivery system. Thus, in the present study, both monolithic nanocomposites from the single-fluid blending process and core-sheath hybrids from the coaxial electrospinning were prepared and utilized as templates to manipulate the self-emulsifying processes. The encapsulation efficiency and drug release profiles were exploited to evaluate the self-assembled emulsions.

## 2 Materials and methods

### 2.1 Materials

Paclitaxel (PTX) was purchased from Shanghai Hao-Sheng Biotechnol. Co. Ltd. (Shanghai, China). Polyvinylpyrrolidone (PVP K90, M = 360,000), polyoxyethylene castor oil (PCO, as emulsifier), and triglyceride (TG, as oil phase) were obtained from Shanghai Aladdin Biochemical Technology Co., Ltd. (Shanghai, China). Anhydrous ethanol was obtained from Sinopharm Chemical Reagent Co., Ltd. (Shanghai, China). Water was double-distilled just before usage.

### 2.2 Electrospinning

Four working fluids were prepared, which are included in Table 1. The first working fluid was a mixed solution in anhydrous ethanol, which contained 8% (w/v) of the polymeric matrix PVP K90, 1.61% (w/v) of the drug PTX, 4% (w/v) of the emulsifier PCO and 2.5% (w/v) of the oil phase TG. A single fluid blending process was exploited to transfer it into solid composite nanofibers, which are denoted as S1 with a drug content of 10% (w/w).

**TABLE 1:** Detailed preparation parameters of nanofibers.

No.	Electro-spinning	Components and compositions (w/v%)		Flow rate (ml/h)		Theoretical drug loading
		Core	Sheath	Core	Sheath	
S1	Blending	PTX 1.61% + PVP 8% + TG 2.5% + PCO 4%	None	1.0	None	10%
S2	Coaxial	PTX 3.22% + PVP 8%	TG 5% + PCO 8% + PVP 8%	0.5	0.5	10%
S3	Coaxial	PTX 5.56% + PVP 8%	TG 5% + PCO 8% + PVP 8%	0.5	1.0	10%

The second sample was prepared using a coaxial electrospinning. A solution containing 3.22% (w/v) of drug and 8% (w/v) of PVP K90 was exploited as a core fluid. A solution containing 8% (w/v) of the emulsifier PCO, 5% (w/v) of the oil phase TG and 8% (w/v) of PVP K90 was exploited as a sheath fluid. The resultant core-shell nanofibers are denoted as S2, whose drug content is 10% (w/w). The third sample was similarly prepared using a coaxial electrospinning. A solution containing 5.56% (w/v) of drug and 8% (w/v) of PVP K90 was exploited as a core fluid. A solution containing 8% (w/v) of the emulsifier PCO, 5% (w/v) of the oil phase TG and 8% (w/v) of PVP K90 was exploited as a sheath fluid. The resultant core-shell nanofibers are denoted as S3, whose drug content is 10% (w/w).

These arrangements are aimed to disclose the influences of two factors (the core-shell structure, and its shell thickness) on the self-emulsifying processes.

The experimental apparatus was a homemade electrospinning system. A homemade concentric spinneret was exploited to conduct both the single-fluid electrospinning for preparing S1 and also the coaxial electrospinning for fabricating S2 and S3. To keep a continuous and stable working process, the applied voltage values during the fabrication was between 12 and 14 kV. The deposition distance was fixed at 15 cm. The environmental parameters included a room temperature of 25°C ± 2°C and a relatively humidity of 51% ± 6%. The collected fibrous films were placed in a dryer to a constant weight, and then were folded and sealed in a self-sealing bag for preservation.

### 2.3 Morphology and inner structure observation of nanofibers

The fibrous films were cut into small patches, which were fixed on the double-sided conductive adhesive. After sprayed with Pt under a nitrogen atmosphere for 60 s, the samples were assessed using a scanning electron microscope (SEM, Quanta FEG-450, FEI corporation, United States). The nanofibers’ sizes were estimated on the SEM images through about 100 places using the ImageJ software (NIH, United States).

The inner structures of the prepared nanofibers S1, S2, and S3 were evaluated using a transmission electron microscope (TEM, JEM 2200-F, JEOL, Japan). The samples were collected by placing the copper grid-supported carbon films above the collectors for 2 min to collect some nanofibers.

### 2.4 Analysis of self-emulsifying properties of electrospun fibers

#### 2.4.1 Drug loading efficiency

A weight of 100 mg fibrous samples was placed into 500 ml water to observe the self-emulsifying processes. The resultant liquids were semitransparent emulsions. The average hydrodynamic diameter and size distribution were assessed using BI-200SM static and dynamic light scattering instruments (SDLC, Brookhaven Instruments Corporation, Austin, Texas, United States).

Meanwhile, a millimeter of the self-assembled emulsion was diluted 10 times, and a drop of the diluted emulsion was dripped on the carbon films supported by 200 mesh copper grids. After naturally dried in the open air, the samples were assessed using the TEM as the above-mentioned manner.

The supernatant of 10 ml self-assembled emulsion was separated from drug-loaded lipid particles by ultra-centrifugation. The free PTX concentration in the supernatant (*Ws*, μg/ml) was measured using a UV-vis spectrophotometer (Unico Instrument Co., Ltd., Shanghai, China) at 228 nm (the maximum absorption peak). A volume of 10 ml emulsion was mixed with 10 ml anhydrous ethanol for releasing all the loaded PTX through demulsification. The general PTX concentration (*Wg*, μg/ml) in the self-emulsified liquids could be determined.

Thereby, the EE values of the self-emulsification processes can be achieved through the following Eq. 1:

$$EE\left(\%\right)=\frac{W_{g}-W_{s}}{W_{g}}\times 100\%$$ (1)

#### 2.4.2 *In vitro* drug release tests

One milliliter of the self-emulsified liquids were placed in a dialysis tube (MWCO = 3,500 Da). An amount of 5.0 mg PTX raw powders were also investigated as a control. The dialysis sac was then dialyzed in 100 ml of phosphate buffer solution (PBS, pH = 6.8, 0.1 M) at a constant temperature of 37°C and a stirring rate of 50 rpm. At predetermined times, the PBS was removed and replaced with the same volume of fresh PBS solution. The amount of drug released was determined by UV-vis spectrophotometer, as mentioned above. Released studies were conducted three times, and mean values were plotted against time.

## 3 Results and discussion

### 3.1 Electrospinning processes and its strategies for self-emulsifying

**FIGURE 2:**
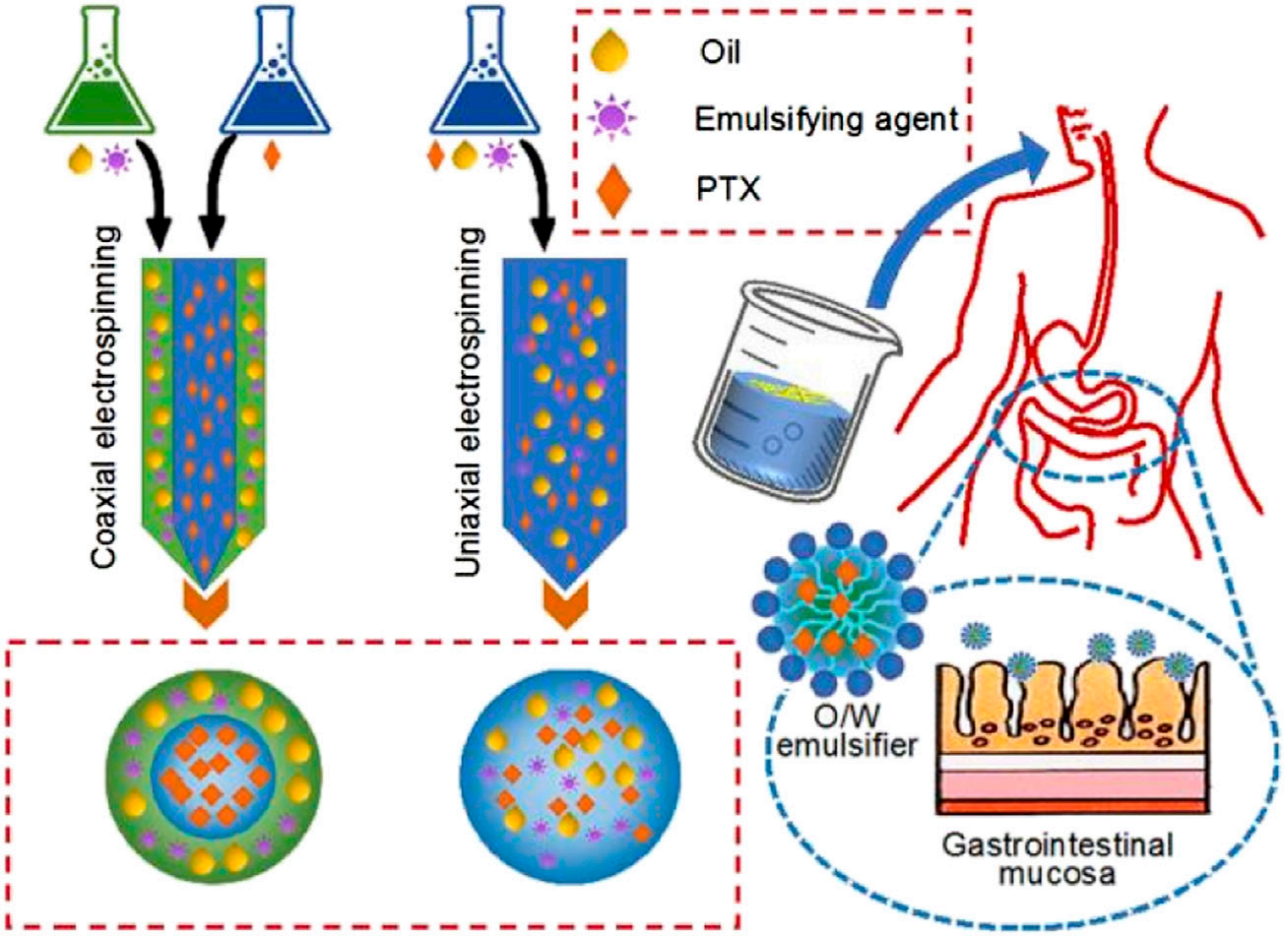
Two strategies from electrospun nanofibers for acting as self-emulsifying drug delivery systems.

In general, SEDDS is an anhydrous pre-nanoemulsion dosage form, which mainly contain isotropic mixtures of oil, surfactant, co-surfactant, drug and polymer molecules traditionally (Chouhan et al., 2015; Nikmaram et al., 2018; Zaghloul et al., 2019). When introduced into aqueous phase of gastric motility, SEDDS is expected to rapidly converted into lipophilic globules, which are often at a nanoscale. Through this way, the solubility and adsorption through gastrointestinal mucosa of a poorly water-soluble drug can be significantly enhanced (Patel and Sawant, 2009; Gao et al., 2020). Electrospinning is able to provide a series of strategies for developing some types of SEDDS. Shown in Figure 2 are diagrams about two different nanofibers-based SEDDS. The first one is the monolithic nanofibers that all the components including drug PTX, emulsifying agent, oil are homogeneously distributed all over the polymeric matrix, which can be easily fabricated from a single-fluid or uniaxial blending electrospinning. The second one is the core-shell nanofibers, in which drug is loaded in the core section, whereas the emulsifying agent and oil are located at the shell section. These components’ arrangement are expected to ensure a better self-emulsifying process.

**FIGURE 3:**
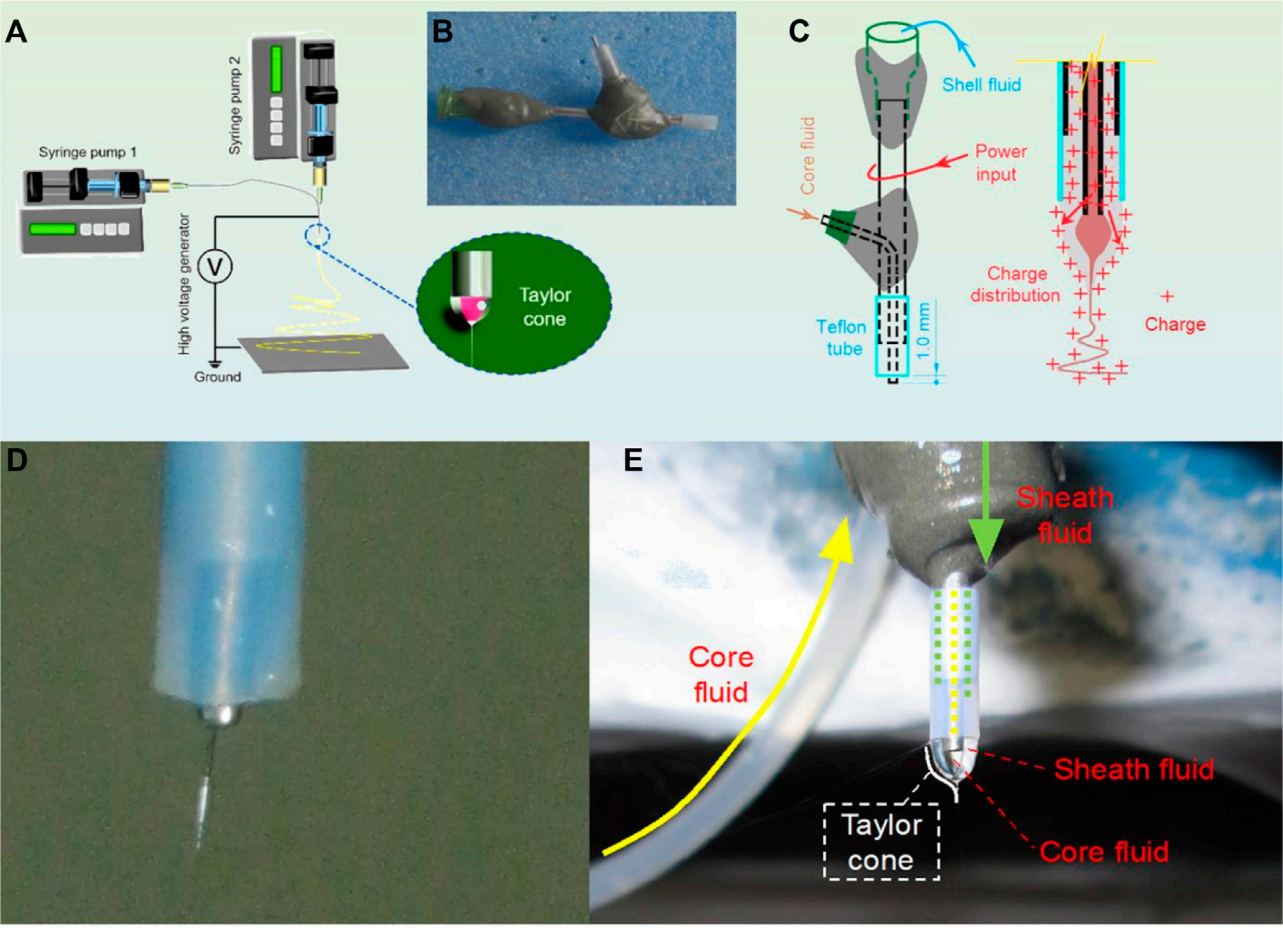
Implementation of the single-fluid and coaxial electrospinning for creating SEDDS: **(A)** A diagram showing the components of an electrospinning system; **(B)** The homemade concentric spinneret; **(C)** The electrohydrodynamic mechanism of ensuring an energy-saving process; **(D)** A typical Taylor cone of the single-fluid blending electrospinning; and **(E)** a coaxial electrospinning process.

Although electrospinning is facile to implement, its process can be improved from several aspects such as the capability of creating complicated nanostructures, production on a large scale, creation of novel functional nanofibers and energy-saving (Kang et al., 2020; Wang et al., 2020; Zhao et al., 2021a; Zhang et al., 2021b; Liu et al., 2022c; Liu et al., 2022d). Shown in Figure 3 are records about the implementations of the single-fluid and coaxial electrospinning in this investigation. The diagram in Figure 3A tells the most fundamental four parts in an electrospinning system, i.e., a spinneret, a grounded collector, a high voltage generator, one or more syringe pumps. The key symbol of the commence of an electrospinning process is the formation of Taylor cone. For coaxial electrospinning, the Taylor cone should be a typical core-shell compound one (as indicated by the bottom-right inset of Figure 3A).

A digital picture about the homemade spinneret is given in Figure 3B. The most significant characteristic is that the spinneret was made from a combination of metal, adhesive (epoxy resin), and plastics. Different with the commercial concentric spinneret, in which all section are made of stainless steel, the homemade spinneret left only a small section of the metal capillary open to the environment for the input of the electrostatic energy. This kind of spinneret was demonstrated to be useful for saving energy. The mechanism is diagrammed in Figure 3C. At the nozzle of spinneret, the charges will move to the surface of shell working fluid naturally to form the compound Taylor cone. During the transportation process, the energy dispersed to the environment will be reduced as smaller as possible.

Figures 3D, E are digital images taken during the preparations of samples S1 and S2, respectively. In Figure 3D, the sheath section had no working fluid, thus, a concentric spinneret was exploited to conduct a single-fluid blending process. In Figure 3E, the double-layer compound Taylor cone could be clearly observed when both core and shell working fluids were pumped to the nozzles of spinneret.

### 3.2 Electrospinning and morphology observation of nanofibers

**FIGURE 4:**
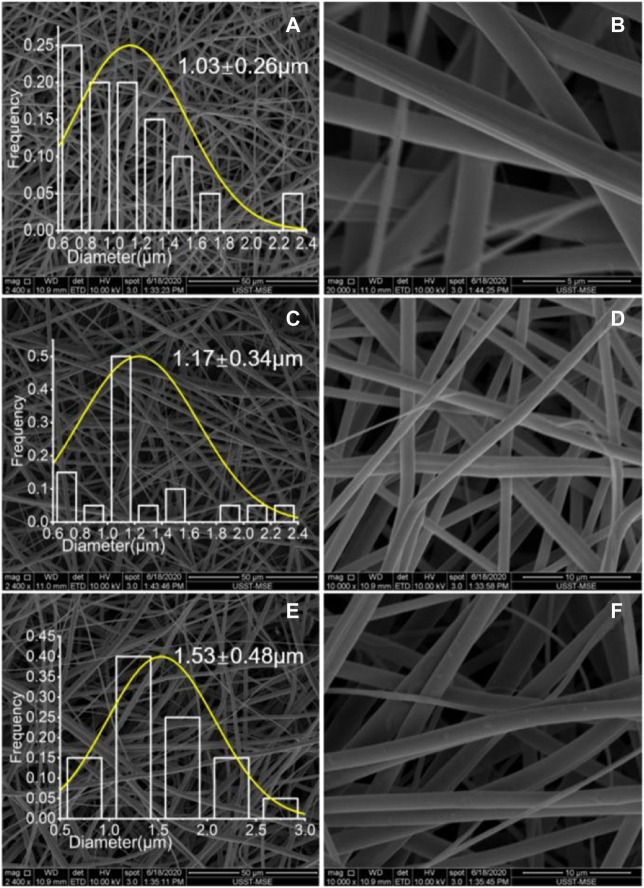
SEM images of the electrospun nanofibers under different magnifications: **(A)** and **(B)** S1; **(C)** and **(D)** S2; **(E)** and **(F)** S3.

Figure 4 shows the SEM images of the three types of nanofibers. All of them have a straight linear morphology with smooth surface. In Figure 4A, the monolithic nanofibers S1 have an average diameter of 1.03 ± 0.26 μm. In Figure 4B, the average diameter of core-shell nanofibers S2 is 1.17 ± 0.34 μm. Both S1 and S2 were fabricated with a total fluid flow rate of 1.0 ml/h. When the total flow rate was increased from 1.0 ml/h to 1.5 ml/h for fabricating nanofibers S3, the applied voltage was also elevated from 12 kV to 14 kV for avoiding the dropping of working fluid on the fiber collector. Meanwhile, the average diameters of the resultant core-shell nanofibers S3 had a significant increase to 1.53 ± 0.48 μm.

**FIGURE 5:**
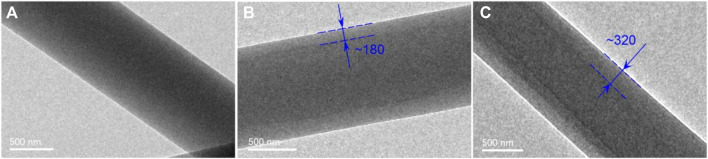
TEM images of the electrospun nanofibers: **(A)** S1; **(B)** S2; **(C)** S3.

The TEM images of the three kinds of nanofibers are included in Figure 5. Just as anticipated, nanofibers S1 from the single-fluid blending electrospinning have a homogeneous structure, as indicated by the similar gray levels all over the nanofibers. In contrast, both nanofibers S2 and S3 have the obvious core-shell structures. By estimations, S2 and S3 fibers have a thickness of 180 and 320 nm, respectively. The increase of sheath fluid flow rate obviously increased the diameters of the whole nanofibers and also the thicknesses of their shell sections.

### 3.3 Analysis of self-emulsifying properties of electrospun fibers

**FIGURE 6:**
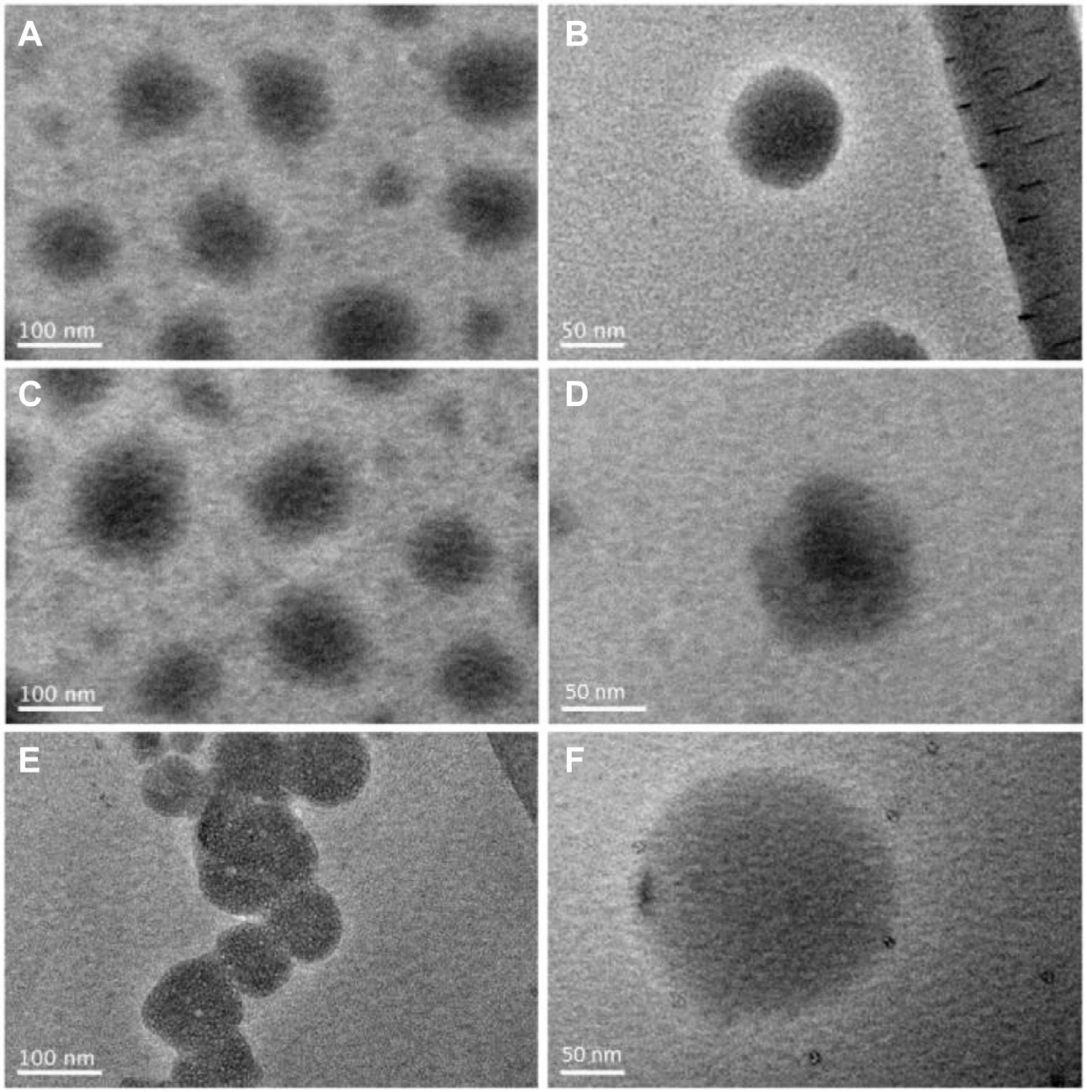
TEM images of the lipid particles suspended in the self-emulsified liquids: **(A)** and **(B)** S1, **(C)** and **(D)** S2, **(E)** and **(F)** S3.

The TEM observations about the nano emulsion particles assembled from the three sorts of nanofibers are included in Figure 6. All the assembled particles have a diameter around 100 nm. Figures 6A, B are particles self-assembled from the homogeneous nanofibers S1 under different magnifications. These particles are round and have a clear boundary, suggesting that the hydrophobic and insoluble components PTX, TG and PCO could aggregate effectively within the nanofibers after the dissolution of the polymeric matrix PVP K90 during the self-emulsifying processes.

Similarly, the particles self-assembled from the core-sheath nanofibers S2 (Figures 6C, D) and those from the nanofibers S3 (Figures 6E, F) have a round morphology with obvious boundaries. Although the “core-sheath” templates (nanofibers S2 and S3) are different with the monolithic composites (nanofibers S1), the self-assembled particles are similar. The intentional distributions of components on the cores-shell nanostructures exhibited no significant influences on the assembled particles’ morphology. However, the real molecular behaviors during the self-emulsifying processes should be different between the monolithic S1 and the core-sheath S2 and S3.

**FIGURE 7:**
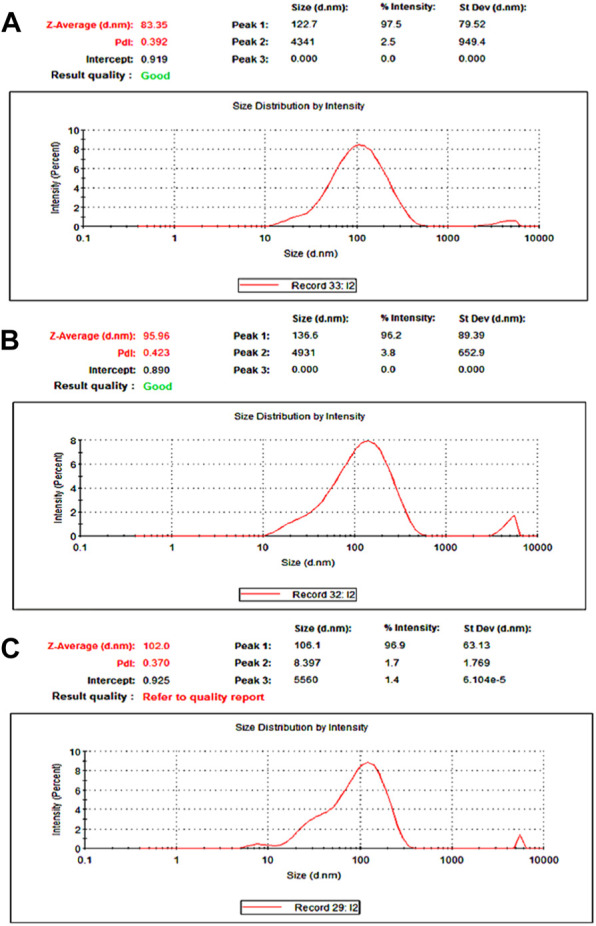
Particle size distribution measured using the laser particle size analyzer **(A)** S1; **(B)** S2; **(C)** S3.

The particle size and size distribution of self-emulsified particles were tested by the dynamic light scattering. The results are included in Figure 7. The average sizes of emulsion particles self-assembled from nanofibers S1, S2, and S3 were 83.35 nm (Figure 7A), 95.96 nm (Figure 7B) and 102.00 nm (Figure 7C), respectively.

It can be seen from Figures 4, 6 that the average diameter of nanofibers and the average particle size of emulsion are positively correlated. That is to say, the smaller the diameter of self-emulsifying fiber, the smaller the emulsion particle size and the better the emulsification results. Two key elements influence the self-emulsification. One is the components that loaded into the nanofibers and their reasonable ratios, which can draw supports from the traditional pharmaceutical knowledge. The other is the nanofibers’ diameter and inner structures. As for controlling the nanofibers’ diameter for a better self-emulsification. The components and their ratios can be kept constant, whereas the fibers’ diameters and inner structures can be manipulated for a better self-emulsifying process.

The small emulsion particles need to pass through the gastrointestinal mucosa for the final adsorption for oral administration. Thus, in general, the smaller the particle size is, the easier it is to be absorbed by the body; and the smaller the particle size, the more the number of particles can pass through, thereby the higher the content of the drug absorbed by the human body. One of the reasons is that when the diameter of the fiber is smaller, its specific surface area is larger, and it can disperse quickly and well when it contacts with water. Another reason is due to the physical and chemical properties of the components themselves, which have an effect on the diameter of the electrospun nanofibers. In this study, the aim is to disclose the influences of different kinds of electrospun inner structures (i.e., monolithic and core-sheath) on the self-emulsifying. As for how to manipulate the diameters of nanofibers, and in turn the size of self-assembled particles, they will be investigated in another study, where modified coaxial and modified triaxial electrospinning with outer fluids of pure solvents will be exploited.

### 3.4 Function performances of the PTX-SEDDS electrospun nanofibers

The standard curve of PTX for UV-vis spectroscopic measurements was A = 2.6801C-0.0137 (R^2^ = 0.99755), where A represents the absorbance of PTX at 228 nm and C represents the PTX concentration in the sample solutions (μg/ml). The measured contents from the self-emulsified emulsions for the nanofibers S1, S2, and S3 are 10.15 ± 0.27%, 9.76 ± 0.42%, and 9.81 ± 0.34%, respectively. These values are closely with the theoretical calculation data, i.e., 10% PTX in the solid nanofibers, suggesting that the electrospinning can completely encapsulate the PTX into the nanofibers, regardless of a single-fluid or a coaxial process. The reason is that the electrospinning is just a physical drying process, and the drying rate is extremely fast. Meanwhile, the drug PTX is stable without sublimation property. Thus, there is no any loss of PTX during the different electrospinning procedures.

After self-emulsifying, 22.1%, 11.6%, and 8.3% of the PTX were dissolved into the supernatant for nanofibers S1, S2, and S3, respectively. These data mean that the EE (%), i.e. the ratio of drug encapsulated into the emulsion particles during self-emulsification, are 77.9%, 88.4% and 91.7% for S1, S2, and S3, respectively. The monolithic nanostructure of S1 released the most PTX to the environment. This is because of the homogeneous distribution of PTX all over the nanofibers S1, which means that there were many PTX molecules were distributed on the surface of S1. These molecules were easy to free into the environment during the formation of emulsion particles during the self-emulsification processes. In sharp contrary, the core-shell nanostructures S2 and S3 have no PTX on the surfaces. All the PTX molecules were located into the core section of nanofibers. Thus, during the self-emulsifying processes, the sheath components TG and PCO are easy to assemble around the PTX to form the emulsion particles. Meanwhile, the thicker the sheath section is, the better the encapsulation effect has. Thus, core-shell fibers S3 has a slightly higher value of 91.7 than S2 of 88.4%.

**FIGURE 8:**
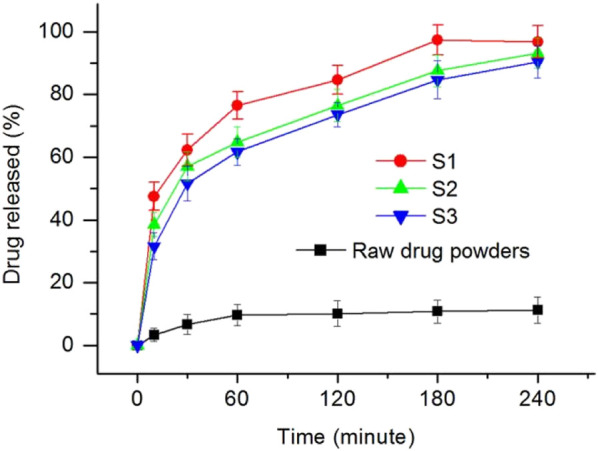
*In Vitro* drug release profiles from the nanofibers S1, S2, S3 and the raw PTX particles.

The results from the *in vitro* dissolution tests are included in Figure 8, in which the raw drug particles’ dissolution behaviors were exploited as a control. During the first 10 min, the drug released contents from the self-emulsified solutions of S1, S2, S3, and PTX powders are 47.5 ± 4.5%, 36.5 ± 3.8%, 31.6 ± 4.3%, and 3.4 ± 2.1%, respectively. Thus, the PTX release ratios from the self-emulsified emulsion particles during the first 10 min were 25.4%, 24.9%, and 23.3% for S1, S2, and S3, respectively (subtracting the sections during self-emulsification, i.e., 22.1%, 11.6% and 8.3%). After 4 h, the PTX released contents for S1, S2, S3 and PTX powders are 96.8 ± 5.2%, 93.2 ± 4.7%, 90.5 ± 5.2%, and 11.2 ± 4.2%, respectively. It can be concluded that, on one hand, the electrospun nanofibers-based SEDDS are able to enhance the dissolution of PTX. On the other hand, the SEDDS are able to provide an extended release profiles for eliminating the possible toxicity resulted from pulsatile release. Meanwhile, the cores-sheath structures S2 and S3 had a better sustained release profiles than the monolithic S1 in terms of initial burst release, and S3 with a thicker sheath layer showed a better result than S2.

In order to reveal the drug release mechanism of PTX from the self-emulsified particles, Peppas Eq. 2 was used to fit the experimental data (Peppas and Narasimhan, 2014).

$$Q\left(t\right)=kt^{n}\;or\;LogQ\left(t\right)=nLogQ\left(t\right)+A$$ (2)

**FIGURE 9:**
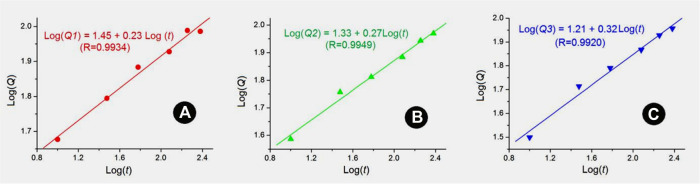
Drug release mechanisms based on Peppas equation: **(A)** S1; **(B)** S2; **(C)** S3.

In the equation, *Q* is the accumulative drug release percentage when the time is *t*, *k*, and *A* are constant values, and the index *n* is an important parameter to indicate the drug release mechanism. When *n* < 0.45, it indicates that the drug is released through the typical Fickian mechanism; when *n* > 0.89, it indicates that the drug is released through the skeleton dissolution mechanism; when *n* is between them, it indicates that the drug is released through the mixed mechanism. It can be seen from Figure 9 that the diffusion indexes of *n* for nanofibers S1, S2 and S3 are 0.23, 0.27, and 0.32 respectively. All these values are smaller than the critical value of 0.45, indicating that the typical Fickian mechanism has played its role in manipulating the PTX molecules release from the self-emulsified particles in the emulsions.

### 3.5 Engineering mechanisms

**FIGURE 10:**
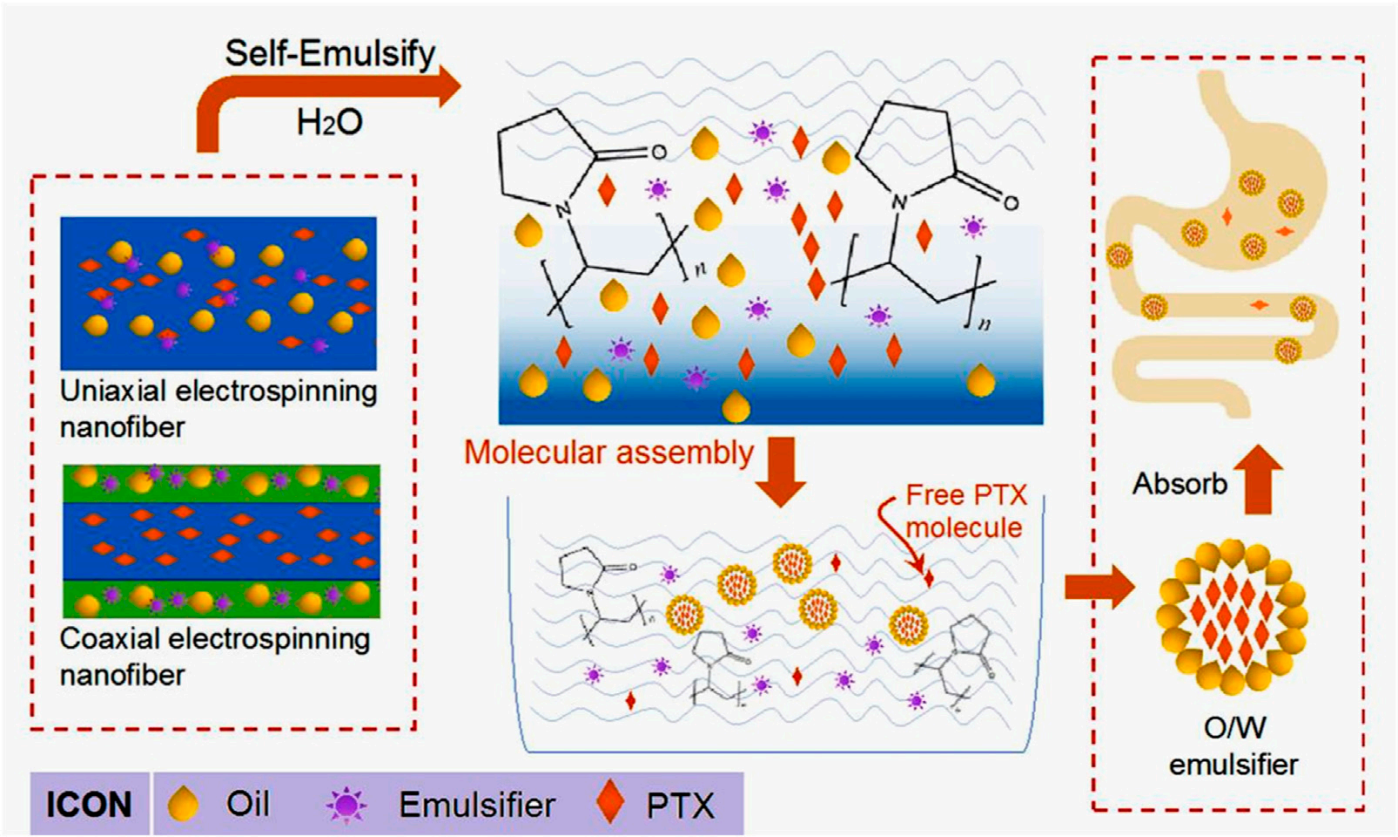
A molecular mechanism of electrospun nanofiber-based SEDDS.

A diagram showing the engineering mechanism on a molecular scale of the electrospun nanofibers-based SEDDS is proposed in Figure 10. Firstly, the electrospinning, both the single-fluid blending process and the coaxial process, is a very fast “top-down” conversion process, by which solutions are converted into solid polymer-based composites. The homogeneous distribution state of components in the working fluids are propagated into the nanofibers. Thus the electrospun nanofibers can be viewed as a mixture on a molecular scale (in pharmaceutics, medicated nanofibers are often called molecular solid dispersion) (Xie et al., 2021; Ziyadi et al., 2021; Afshar et al., 2022; Zhou et al., 2022).

When these nanofibers are placed into water, the soluble polymeric matrix will absorb water, swell, disentangle, and free into the bulk solution. During these processes, the anchored insoluble molecules such as PTX, PCO and TG will free from the polymeric restriction but within the confined region regulated by the nanofibers’ diameter. The hydrophobic interactions between themselves and from the surrounding water molecules will promote the self-aggregation to form the emulsion particles. These emulsion particles not only enhance the dissolution of PTX, but also benefit a useful trans-membrane for adsorption with smaller gastrointestinal irritation. The cytotoxicology tests and animal experiments of these nanofiber-based SEDDS will be further conducted in future.

In this nano era, more and more “top-down” and “bottom-up” techniques are developed for bioengineering and biotechnology, accompanied with a series of novel functional materials such as hydrogels and new types of polymers (Li et al., 2022b; Zhang et al., 2022b; Wang et al., 2022c; Wang et al., 2022d Song et al., 2022; Wang and Feng, 2022; Zhu et al., 2022) and even inorganic nanoparticles (Tang et al., 2022b; Chen et al., 2022c; Wu et al., 2022). The present protocols showed a frontier, in which the combination of a top-down electrospinning and a bottom-up of molecular self-emulsifying was explored to fabricate biomedical materials for resolving one of the most difficult challenges, i.e. the therapeutic delivery of poorly water-soluble drugs.

## 4 Conclusion

In this study, PVP K90 was used as a filament-forming matrix and meanwhile a carrier of oil phase, emulsifier and insoluble model drug for preparing SEDDS using both a single-fluid blending and two coaxial electrospinning processes. Electrospun monolithic nanofibers S1 and core-sheath nanofibers S2 and S3 were loaded with a fixed PTX content of 10%. These nanofibers are demonstrated to have the linear morphology and homogeneous or cores-sheath structures through the assessments of SEM and TEM. The TEM and SDLC experiments were conducted to characterize the self-emulsified emulsions. The particles’ sizes self-assembled from S1, S2, and S3 are 83.35 nm, 95.96 and 102.00 nm, respectively. The evaluation of EE and *in vitro* dissolution tests suggested that the functional performances have an order of S1 ˂ S2 ˂ S3. A core-sheath structure is a better template for manipulating self-emulsifying than the monolithic nanofibers. Meanwhile, the thickness of the sheath section in the core-sheath structure can generate a positive influence on the self-emulsified emulsions, which can be facilely tailored by the sheath fluid flow rate during the preparation process. The protocols reported here pave a new way for effective oral delivery of poorly water-soluble drug.

## Data Availability

The original contributions presented in the study are included in the article/Supplementary Material, further inquiries can be directed to the corresponding authors.
